# Supplementary material for: Hybrid Models and Biological Model Reduction with PyDSTool
Source: PLoS Comput Biol. 2012 Aug 9;8(8):e1002628. doi: 10.1371/journal.pcbi.1002628 (PMC3415397; doi:10.1371/journal.pcbi.1002628)
Supplement: Text S4 — Complete source code for the PyDSTool package (version 0.88.120504). Includes API documentation and help files linking to web pages. This file is identical to the current public release on Sourceforge.net. (ZIP) [file pcbi.1002628.s004.zip › PyDSTool/html/toc-PyDSTool-module.html]

xml version="1.0" encoding="ascii"?


PyDSTool


# Module PyDSTool

---

## Functions

loadSession  
 restart  
 saveSession  
 who  

## Variables

ALLOW\_THREADS  
 Abs  
 Acos  
 Asin  
 Atan  
 Atan2  
 BUFSIZE  
 Betavariate  
 CLIP  
 Ceil  
 Choice  
 Cos  
 Cosh  
 Degrees  
 E  
 ERR\_CALL  
 ERR\_DEFAULT  
 ERR\_DEFAULT2  
 ERR\_IGNORE  
 ERR\_LOG  
 ERR\_PRINT  
 ERR\_RAISE  
 ERR\_WARN  
 Exp  
 Expovariate  
 FLOATING\_POINT\_SUPPORT  
 FPE\_DIVIDEBYZERO  
 FPE\_INVALID  
 FPE\_OVERFLOW  
 FPE\_UNDERFLOW  
 Fabs  
 False\_  
 Floor  
 Fmod  
 Frexp  
 Gammavariate  
 Gauss  
 Getrandbits  
 Getstate  
 Hypot  
 Infinity  
 Jumpahead  
 Ldexp  
 Log  
 Log10  
 Lognormvariate  
 MAXDIMS  
 Max  
 Min  
 Modf  
 NAN  
 NINF  
 NZERO  
 Normalvariate  
 PINF  
 PZERO  
 Paretovariate  
 Pi  
 Pow  
 RAISE  
 Radians  
 Randint  
 Random  
 Randrange  
 SHIFT\_DIVIDEBYZERO  
 SHIFT\_INVALID  
 SHIFT\_OVERFLOW  
 SHIFT\_UNDERFLOW  
 Sample  
 ScalarType  
 Seed  
 Setstate  
 Shuffle  
 Sin  
 Sinh  
 Sqrt  
 Sum  
 Systemrandom  
 Tan  
 Tanh  
 True\_  
 UFUNC\_BUFSIZE\_DEFAULT  
 UFUNC\_PYVALS\_NAME  
 Uniform  
 Vonmisesvariate  
 WRAP  
 Weibullvariate  
 Wichmannhill  

\_1DimplicitSolveMethods

\_\_LICENSE\_\_  
 \_\_revision\_\_  

\_\_session\_ext

\_\_symbolic\_ext

\_implicitSolveMethods

\_pyDSToolTypes

absolute  
 add  
 bitwise\_and  
 bitwise\_not  
 bitwise\_or  
 bitwise\_xor  
 c\_  
 cast  
 conj  
 conjugate  
 copysign  
 deg2rad  
 divide  
 equal  
 exp2  
 expm1  
 floor\_divide  
 fmax  
 fmin  
 greater\_equal  
 index\_exp  
 inf  
 infty  
 invert  
 isinf  
 left\_shift  
 little\_endian  
 log1p  
 logaddexp  
 logaddexp2  
 logical\_and  
 logical\_not  
 logical\_xor  
 maximum  
 mgrid  
 minimum  
 multiply  
 n  
 nan  
 nbytes  
 negative  
 newaxis  
 nextafter  
 not\_equal  
 ogrid  
 ones\_like  
 r\_  
 rad2deg  
 reciprocal  
 remainder  
 right\_shift  
 rint  
 s\_  
 sctypeDict  
 sctypeNA  
 sctypes  
 signbit  
 spacing  
 square  
 subtract  
 t  
 true\_divide  
 trunc  
 typeDict  
 typeNA  
 typecodes  
 verstr\_parts  

---

[hide private]
